# Role of Gut Microbial Metabolites in Cardiovascular Diseases—Current Insights and the Road Ahead

**DOI:** 10.3390/ijms251810208

**Published:** 2024-09-23

**Authors:** Sayantap Datta, Sindhura Pasham, Sriram Inavolu, Krishna M. Boini, Saisudha Koka

**Affiliations:** 1 Department of Pharmacological and Pharmaceutical Sciences, College of Pharmacy, University of Houston, Houston, TX 77204, USA; 2 Department of Pharmaceutical Sciences, Irma Lerma College of Pharmacy, Texas A&M University, Kingsville, TX 78363, USA

**Keywords:** gut microbial metabolites, cardiovascular diseases, short-chain fatty acids, TMAO

## Abstract

Cardiovascular diseases (CVDs) are the leading cause of premature morbidity and mortality globally. The identification of novel risk factors contributing to CVD onset and progression has enabled an improved understanding of CVD pathophysiology. In addition to the conventional risk factors like high blood pressure, diabetes, obesity and smoking, the role of gut microbiome and intestinal microbe-derived metabolites in maintaining cardiovascular health has gained recent attention in the field of CVD pathophysiology. The human gastrointestinal tract caters to a highly diverse spectrum of microbes recognized as the gut microbiota, which are central to several physiologically significant cascades such as metabolism, nutrient absorption, and energy balance. The manipulation of the gut microbial subtleties potentially contributes to CVD, inflammation, neurodegeneration, obesity, and diabetic onset. The existing paradigm of studies suggests that the disruption of the gut microbial dynamics contributes towards CVD incidence. However, the exact mechanistic understanding of such a correlation from a signaling perspective remains elusive. This review has focused upon an in-depth characterization of gut microbial metabolites and their role in varied pathophysiological conditions, and highlights the potential molecular and signaling mechanisms governing the gut microbial metabolites in CVDs. In addition, it summarizes the existing courses of therapy in modulating the gut microbiome and its metabolites, limitations and scientific gaps in our current understanding, as well as future directions of studies involving the modulation of the gut microbiome and its metabolites, which can be undertaken to develop CVD-associated treatment options. Clarity in the understanding of the molecular interaction(s) and associations governing the gut microbiome and CVD shall potentially enable the development of novel druggable targets to ameliorate CVD in the years to come.

## 1. Introduction

Cardiovascular diseases (CVDs) constitute the most frequent known non-communicable diseases, accounting for an approximately 17.9 million mortalities globally [1,2]. Several risk factors play a pivotal role in CVD onset and progression, including hypertension, tobacco smoking, unrestrained alcohol consumption, dyslipidemia, diabetic incidence and a sedentary lifestyle [3,4]. CVDs encompass a broad spectrum of cardiovascular dysfunctions that chiefly include coronary heart disease [5,6], cerebrovascular anomalies [7,8], peripheral arterial dysfunction [9,10], rheumatic cardiac malfunction [11,12], congenital heart defects [13,14], myocardial infarction (MI) [15,16,17] and pulmonary embolism [18,19]. The commonality among these dysfunctions essentially results in defects in cardiac morphogenesis, repair, and cardiac functioning rhythm [17,20]. The heart encounters a marked decrease in proliferative cardiomyocyte count upon CVD onset. Adult cardiomyocytes exhibit a compromised ability to proliferate, which limits the cardiac regenerative capacity, accounting for ventricular remodeling, a loss in muscle strength, contractility and subsequent congestive heart failure [21,22]. Although significant progress has been made in CVD therapy development [23,24], it is important to recognize the emerging risk factors and novel molecular signaling cascades governing CVD pathophysiology.

The identification of the role of risk factors in contributing towards CVD onset and progression has enabled a better understanding of the plethora of biochemical and histological cascades driving CVD onset and progression, characterized by vascular and endothelial dysfunction [25,26], inflammatory episodes [27,28], prothrombotic status [29,30], impact on basal metabolism [31,32] and lipid profile imbalance [33,34]. Observational cohort studies and clinical trials emphasize the persistence of residual inflammatory risk in CVD patients post statin therapy [35,36,37]. Although precision medicine-based studies hold great promise for improved, personalized, integrated and patient-specific approaches for CVD therapy, patient-centric needs demand the identification of new risk factors and druggable targets for better outcomes of CVD therapy in cardiac patients [38]. In this context, gut microbiome and their metabolites have recently been recognized as novel risk factors affecting a variety of physiological processes and resulting in several disease pathologies, including CVDs.

The human gastrointestinal tract accommodates a diverse and dynamic spectrum of the microbial population, collectively referred to as gut microbiota [39,40]. It extends across the intestinal epithelium and is chiefly composed of physiologically beneficial populations of *Firmicutes*, *Bacteroidetes*, *Actinobacteria*, *Proteobacteria*, and *Verrucomicrobia* [41,42,43]. The advent of state-of-the-art techniques such as metagenomic analysis, marker gene analysis, and meta-transcriptome have enabled an improved characterization of the gut microbiota [44,45,46]. From a functional perspective, the gut microbiota primarily develop and maintain the intestinal barrier, the activation of the immune system and the metabolic breakdown of nutrients and drugs [47,48,49]. At the endocrine level, the gut microbiota modulates the metabolism of leptin, ghrelin, and cortisol [50]. Dysbiosis of the gut microbial dynamics, caused due to anomalies in diet, enteric pathogen population, toxins, and other environmental factors, results in diverse ailments like inflammatory bowel disease, diabetes, allergic onset, and neurological complications [51,52,53,54]. The gut microbial breakdown of dietary food components generates a broad spectrum of metabolites, such as short-chain fatty acids (SCFA), branched-chain fatty acids, amines, phenolic compounds, hydrogen sulfide, and methane [55,56,57,58,59,60]. Studies have proven that these gut microbial metabolites play a pivotal role in affecting cardiovascular functioning [61,62,63]. The gut microbiome and the heart interact with each other through these metabolites that are reabsorbed and subsequently transferred to systemic circulation [64,65]. Certain gut-microbe-derived-metabolites are beneficial to cardiac functioning, while some have been reported to be detrimental [65,66]. Although a cause–effect relationship between gut microbial metabolites and cardiovascular anomalies has been reported, the exact signaling mechanism governing such interactions remains unclear.

This review focuses on the signaling mechanisms identified so far, that drive the interaction between gut microbial metabolites and cardiovascular function. The metabolites that play a pivotal role in the CVD onset and progression and the status of their druggability is comprehensively discussed. An in-depth understanding of the signaling cross-talks involved in the gut–heart axis shall potentially benefit towards the development of novel and effective druggable targets to ameliorate CVD over the long term.

## 2. Pathophysiology of Gut Microbial Metabolites in CVDs

The gut microbial/host metabolic interactions play a pivotal role in the generation of a broad spectrum of metabolites and associated small molecules [67,68,69]. The extent of the generation of these metabolites is usually determined by interindividual genomic differences and the availability of dietary substrates, among multiple other environmental factors [70,71,72]. These metabolites undergo absorption across the host gut, enter systemic body circulation and influence vital physiological processes [68,73].

### 2.1. Short-Chain Fatty Acids (SCFAs)

Short-chain fatty acids (SCFAs) constitute a majority of the gut microbial metabolites produced because of the gut microbial fermentation of dietary macromolecules. The gut microbiota participate in the metabolic breakdown of sugars via the saccharolytic pathway and protein fermentation to generate SCFAs [74]. Protein fermentation additionally contributes towards the generation of co-metabolites like amines, ammonia, thiols, indoles, and other polyphenolic compounds [75]. The SCFAs exercise tissue-specific physiological influence, primarily driven by their concentration differences in the gut lumen and circulating blood, as well as the hepatic and peripheral sites [76,77]. Functionally, the SCFAs contribute towards the prevention of the onset and progression of metabolic dysfunctions, inflammatory complications, and cancer [78,79]. SCFAs inhibit the formation of foam cells and the influx of monocytes, as well as reducing the production of pro-inflammatory cytokines by the endothelium, and thus contribute to the recovery of endothelial dysfunction. SCFAs protect against obesity, improve glucose tolerance, and attenuate ulcerative colitis, Crohn’s disease, and antibiotic-associated diarrhea [80,81]. SCFAs augment postprandial plasma peptide YY (PYY) and glucagon-like peptide-1 (GLP-1) release from colon cells and downregulate intra-abdominal adipose tissue distribution and intrahepatocellular lipid content [82].

From a molecular perspective, SCFAs mediate their protective functions predominantly via the activation of the SCFA receptor-G-protein-coupled receptor 43 (GPR43), also identified as free fatty acid receptor 2 (FFAR2) [83]. GPR43 is chiefly expressed in the intestinal endocrine L-cells and its activation by SCFAs augments GLP-1 secretion [84]. SCFA-induced GPR43 activation also accounts for the decrease in adipose-specific insulin signaling [85]. This, in turn, hinders fat accumulation and attenuates obesity [86]. Studies have also shown that SCFA-induced GPR43 activation strengthens the colonic T cell population and exhibits a protective function against colitis, and type 2 diabetes [87]. SCFAs also activate GPR41, also known as FFAR3, in the peripheral nerves, and improve insulin resistance [88]. GPR41 activation by SCFAs increases macrophage and dendritic cell precursors and protects against respiratory problems [89]. The SCFA-associated activation of the colonic epithelium localized GPR109A receptor promotes anti-inflammatory factors in colonic macrophages, and augments the differentiation of regulatory and interleukin (IL)-10 producing T cells. This, in turn, prevents colonic inflammation and subsequent cancer incidence [90,91,92,93]. Thus, SCFAs activate multiple GPRs and mediate a variety of physiological and pathological processes [94].

### 2.2. Bile Acids

Bile acids (BAs) are amphipathic molecules generated in the liver from cholesterol and stored in the gall bladder [95]. The overall composition of the gut microbiota and associated influence on BA biogenesis are key drivers of interspecies and interindividual BA pool heterogeneity [96,97]. The mammalian BA pool comprises primary BA and secondary BA. Primary BAs are generated by hepatocytes and stored in the gall bladder, while the secondary BAs are primarily derived from gut microbial metabolism [98]. Primary BAs are either produced by cholesterol-7α-hydroxylase (CYP7A1) via the classical pathway in hepatocytes or by 27-hydroxylase (CYP27A1) via the acidic pathway in extrahepatic sites [98]. The primary BAs undergo conjugation with glycine or taurine and form bile salts and secondary BAs to be stored in gall bladder [99]. BAs determine the overall metabolic capacity of the gut microbiota. Primary BAs upregulate the expression of BA-metabolizing genes in the small intestine [100,101]. Excessive amphipathic BAs induce DNA damage, protein misfolding and oxidative stress, which can lower gut bacterial viability [102,103]. However, BAs also exhibit a protective role towards gut microbial diversity [100]. This is proven by studies demonstrating that cholestasis downregulates gut microbial diversity and functioning [104]. BAs potentially serve as energy sources to accommodate the needs to sustain gut microbial diversity [104].

**Figure 1:**
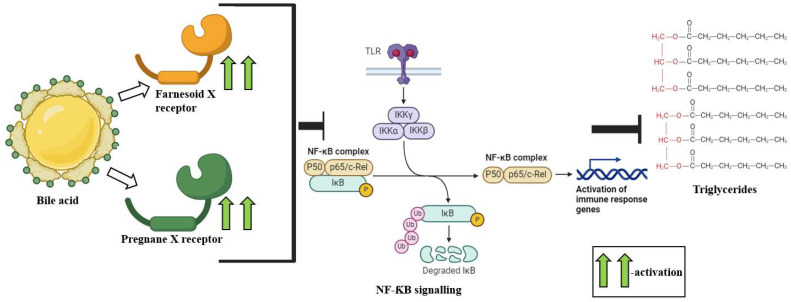
A schematic representation of the protective role of BAs in hypertriglyceridemia. The BA-associated activation of the Farnesoid X receptor and Pregnane X receptor attenuates pro-inflammatory NF-κB signaling and downregulates triglyceride levels.

From a pathophysiological perspective, BAs directly impede the differentiation and activation of the *Clostridium difficile* infection [105]. The upregulation of primary BA levels and downregulation of secondary BAs have been reported in inflammatory bowel disease (IBD), primarily via the suppression of nuclear factor Kappa-light-chain-enhancer of activated B cells (NF-κB) via the Farnesoid X receptor (FXR) and Pregnane X receptor (PXR) activation [106,107,108]. BAs also exhibit a beneficial impact against hypertriglyceridemia by the activation of the Farnesoid X receptor and Pregnane X receptor, which attenuates pro-inflammatory NF-κB signaling and downregulates triglyceride levels [94] (Figure 1). BA-associated FXR/SHP activation intervenes in the fatty acid biosynthesis driven by the liver X receptor (LXR) and sterol regulatory element-binding protein 1c (SREBP-1c) [109,110]. Secondary BAs have also been reported to ameliorate metabolic syndrome, although the mechanism governing the process remains unclear [111]. The gut-microbiota-derived BAs have been reported to augment hepatocellular carcinoma incidence by increasing the levels of deoxycholic acid (DCA) by 7α-hydroxylases [112,113]. Secondary BAs have been reported to exhibit greater carcinogenic potential as compared to primary Bas, mainly because of increased hydrophobicity, the disruption of the cellular membrane and the subsequent induction of cell damage responses [113,114]. Increased secondary BAs also augment reactive oxygen species (ROS) levels via phospholipase A_2_ activation [115,116].

### 2.3. Trimethylamine N-Oxide (TMAO)

The gut microbiota generate trimethylamine N-oxide (TMAO) from dietary products, employing microbial trimethylamine (TMA) lyases [117,118]. The gut-microbiota-driven metabolic conversion of choline, phosphatidylcholine and L-carnitine to TMA is catalyzed by TMA lyase, which breaks the carbon–nitrogen bonds in nutrient macromolecules and generates TMA [119,120]. TMA enters systemic circulation through the vessel wall, reaches the liver via hepatic portal circulation and is subsequently oxidized to TMAO by hepatic flavin monooxygenase-3 (FMO3) [121,122]. The generated TMAO re-enters systemic circulation and eventually accumulates in body tissues [123]. Plasma TMAO levels in healthy humans usually remain less than or equal to 5 µM and can shoot up to about 40 µM in patients with renal damage [124]. Other factors influencing TMAO levels in the body include age, cholic acid intake in food, and sex hormone levels [125,126]. Although kidneys are the primary organs involved in the TMAO elimination from the body, TMAO can also be eliminated via sweat, respiration, or fecal matter [127,128].

**Figure 2:**
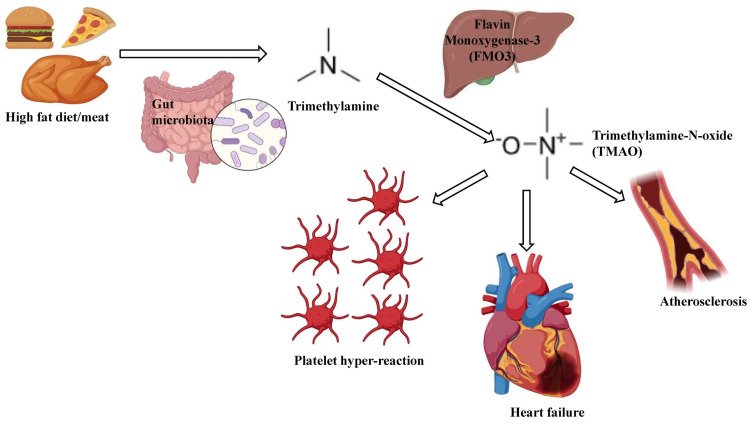
Schematic representation of TMAO generation and associated pathophysiological correlations. High fat dietary intake leads to trimethylamine (TMA) generation upon gut microbial breakdown. TMA undergoes oxidization via hepatic flavin monooxygenase-3 (FMO3) and produces TMAO. This TMAO correlates with varied pathophysiological onset viz atherosclerosis, heart failure and platelet hyper-reactivity.

Recent studies have shown that TMAO exhibits a large pathophysiological influence on the onset and progression of heart failure, chronic kidney diseases, atherosclerosis, obesity, and diabetes [129,130,131,132]. Increased TMAO facilitates atherosclerotic plaque formation [122]. TMAO also augments thrombotic risk, promoting arterial thrombosis formation and shortening the duration of vascular occlusion [133]. It has been reported that TMAO promotes thrombin, collagen, platelet hyperactivity, endogenous calcium release from platelets, and amplified platelet adhesion to collagen [133]. TMAO also promotes monocyte adhesion to endothelial cells during foam cell formation and increases the count of scavenger receptors on macrophages [134,135]. Increased TMAO levels alter the static electricity at the arterial wall and significantly affect the inflow/efflux of fatty deposits on the arterial wall [136]. Consequently, foam cell formation rises and culminates to atherosclerotic plaque formation [136]. TMAO also has significant impact on cholesterol metabolism by reducing reverse cholesterol transport (RCT) [137]. This increases blood cholesterol levels and aggravates atherosclerotic progression [138]. Thus, TMAO is involved in multiple pathophysiological processes, including atherosclerosis, heart failure and platelet hyperreactivity [94] (Figure 2).

### 2.4. Indole-3-Propionic Acid (IPA)

Indole-3-propionic acid (IPA) is a metabolite of the gut-microbiota-driven breakdown of dietary tryptophan [139]. Serum IPA levels usually vary between 1 and 10 µM in humans under homeostatic conditions [140,141]. The gut microbial population predominantly involved in IPA formation include *Lactobacillus reuteri*, *Clostridium sporogenes*, *Clostridium caloritolerans* and members of *Peptostreptococci* genus [142,143]. The gut-microbiota-derived IPA production is chiefly governed by tryptophan aminotransferase [144,145,146]. Metabolic studies reveal that IPA augments gut–blood barrier functions by promoting the expression of claudins and tight junction proteins [147,148]. IPA is also involved in the activation of aryl hydrocarbon receptor in colonic epithelium and culminates in anti-inflammatory and anti-cancer effects [149,150]. IPA also exhibits a protective function against oxidative stress and lipid peroxidation damage [151,152,153]. IPA attenuates hematopoietic and gastrointestinal side effects in breast cancer cells [154,155]. From a neurological perspective, IPA downregulates the accumulation of amyloid β-proteins and restores mitochondrial functionality in Alzheimer’s disease [156,157]. IPA also prevents oxidative-stress-induced brain damage and ROS-induced cell death in Parkinson’s disease [158,159].

### 2.5. Hydrogen Sulfide

Gut-microbiota-driven hydrogen sulfide (H_2_S) is primarily generated from sulfate-reducing bacteria in the gut, which mainly include *Desulfovibrio piger*, *Desulfovibrio desulficans* and bacteria belonging to the *Desulfobulbus* and *Desulfotomaculu* genera [160,161]. These bacteria usually need a sulfate substrate and an electron donor for the generation of H_2_S [162]. Apart from these sulfate-reducing bacteria, anaerobic bacterial groups like *Escherichia coli*, *Salmonella enterica* and *Enterobacter aerogenes* mediate the catalytic breakdown of sulfur-containing cysteine into H_2_S, pyruvate and ammonia via cysteine desulfhydrase [163,164]. Sulfite reductase, a component of *Escherichia coli*, *Salmonella* and *Klebsiella*, also mediates H_2_S production via sulfite reduction reactions [165,166,167,168]. H_2_S acts as a signaling molecule that induces free radical responses and strengthens gut bacterial resistance to antibiotics [169]. H_2_S improves colon barrier integrity and downregulates local and systemic inflammatory cascades via the modulation of macrophages and cluster of differentiation (CD)8^+^ T cells [170,171]. H_2_S also prevents Nucleotide-binding oligomerization domain Leucine-rich Repeat and Pyrin domain-containing 3 (NLRP3) inflammasome-driven neuroinflammation via purinoreceptor-7 (P2X7) 14 inhibition in immune cells [172,173,174].

### 2.6. Phenylacetylglutamine (PAGln)

Phenylalanine, an essential amino acid, constitutes a chief precursor towards the synthesis of phenylacetylglutamine (PAGln)—primarily mediated via the meta organismal signaling pathway [175,176]. Unabsorbed phenylalanine culminates in the generation of phenylacetic acid (PAA) [177]. PAA, upon intestinal absorption, interacts with available glutamine residues and forms PAGln—chiefly catalyzed by hepatic enzymes [176].

Non-targeted metabolomic studies have established a strong correlation between PAGln and CVD onset, especially in type-2 diabetes patients [178]. PAGln has been reported to influence platelet functioning [179]. Studies have also reported that PAGln augments platelet adhesion to the collagen matrix and intracytoplasmic Ca^2+^ concentration [180,181]. The structural similarity with catecholamines has also led to hypotheses that PAGln can potentially modulate adrenergic receptors, accounting for GPCR (α2A, α2B, β2)-induced platelet reactivity and thrombosis [175]. This has been further substantiated via animal model studies, whereby PAGln was reported to trigger platelet activation and thrombosis—primarily driven via adrenergic signaling [178]. Patient-centric studies have reported that PAGln levels in plasma were significantly raised in coronary artery disease (CAD) patients with stent stenosis and hyperplasia [182]. This consolidates the idea that a rise in bacterial PAGln synthase-induced circulating PAGln accounts for in-stent stenosis in CAD patients [176].

## 3. Molecular Mechanisms of Gut Microbial Metabolites in Cardiovascular Diseases

Dysbiosis of the gut microbiota largely contributes towards the onset and progression of CVDs [183]. Heart failure (HF) patients exhibit a rise in *Bifidobacterium* and *Enterobacteriaceae* populations and a decreased count of *Faecalibacterium prausnitzii* [184,185,186]. Compromised gut microbial dynamics promote intestinal permeability and culminates in a leaky gut, prone to inflammation [187,188]. Gut microbial dysbiosis is attributed to diastolic dysfunction and augments left ventricular hypertrophy [189,190]. The gut microbiota interferes with the cardiac functioning through metabolites that undergo intestinal absorption and enter systemic circulation [191]. These metabolites have intrinsic signaling cross-talks in driving the gut microbiota/heart interaction axis, and contribute to CVDs.

### 3.1. Short-Chain Fatty Acids (SCFAs)

**Figure 3:**
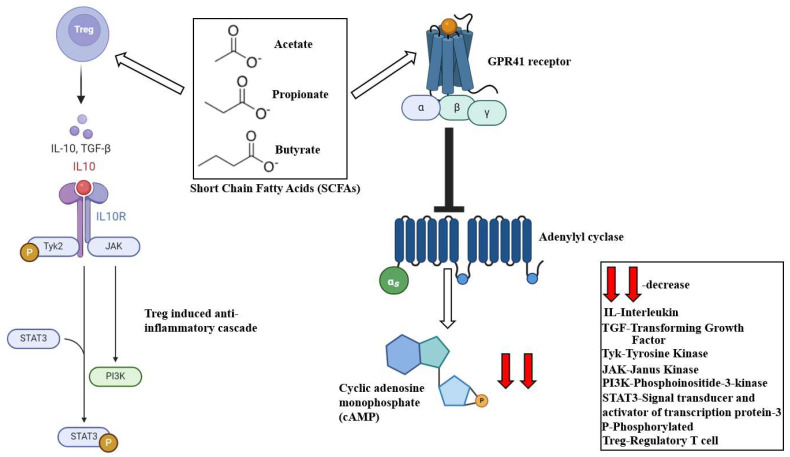
Schematic representation of SCFA-mediated cardioprotective signaling influence. SCFA-mediated GPR41 activation deactivates adenylyl cyclase and attenuates cAMP release, ameliorating blood pressure anomalies. SCFAs also augment regulatory T cell (Treg)-associated anti-inflammatory IL-10 signaling and attenuate cardiovascular injury.

Several studies established that gut microbial derived SCFAs are central to therapeutic potential against hypertension [192]. SCFAs downregulate cardiac hypertrophy, fibrosis and associated vascular dysfunction through regulatory T cell (Treg) modulation [193]. SCFAs also play a central role in decreasing luminal pH and mucus production and the subsequent maintenance of the gut barrier integrity [183]. SCFAs exercise anti-inflammatory functions and modulate lipid metabolism and gluconeogenesis [194]. SCFAs block cholesterol generation and hepatic localization. These eventually culminate in a long-term impact on cardiometabolic functioning, thereby inhibiting the onset of coronary diseases [194]. The alteration of the gut microbiome dynamics interferes with SCFA production and is attributed to CVD pathophysiology. A decrease in SCFA production compromises its anti-inflammatory functions and culminates in atherogenesis [195]. Accelerated inflammatory cascades lead to cardiovascular injury via the downregulation of Treg-induced IL-10 production [196,197]. SCFA supplementation promotes Treg-associated anti-inflammatory response and reduces the local infiltration of immune cells [193]. This downregulates susceptibility to cardiac arrhythmia and atherosclerotic lesion formation [198]. SCFAs also reverse cardiomyocyte contraction anomalies by promoting the expression of protein phosphatases, endothelial nitric oxide synthase (eNOS), phosphorylated phosphatase and TENsin homologue (PTEN) and glycogen synthase kinase-3β (GSK-3β) [93]. In regulating blood pressure, SCFAs modulate GPR41 and olfactory receptor-78 (OLFR78) receptors [93]. GPR41 is typically a Gαi/o-coupled receptor. SCFA-induced GPR41 activation inhibits the formation of cyclic adenosine monophosphate (cAMP), protein kinase A [94] (Figure 3) and the activation of p38 and extracellular signal-regulated kinase (ERK) [199,200]. The activation of the olfactory receptors leads to an increase in cAMP and Ca^2+^ production [201,202].

### 3.2. Trimethylamine-N-Oxide (TMAO)

**Figure 4:**
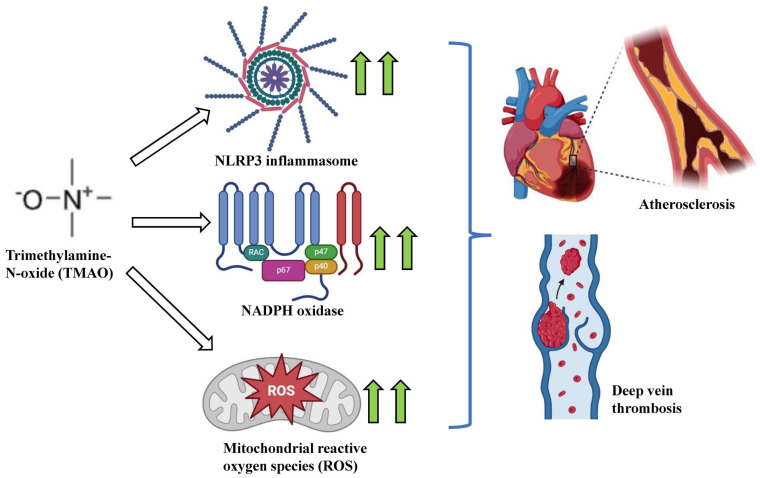
Schematic representation of TMAO-driven CVD risk. TMAO increases NLRP3 inflammasome activity, mitochondrial ROS levels and NADPH oxidase activity and culminates in CVD onset viz atherosclerosis and deep vein thrombosis.

The gut microbiome also promotes atherosclerosis onset and progression through the enhanced production of TMAO [203]. Gut bacteria, primarily, *Deferribacteraceae*, *Anaeroplasmataceae*, *Prevotellaceae*, and *Enterobacteriaceae*, contribute towards TMAO formation [204]. TMAO causes vascular injury through the modulation of cholesterol metabolism, promoting ROS production, augmenting vascular endothelial damage, cell junction rupture and an increase in cellular permeability [205,206,207]. Increased TMAO levels promote the adhesion of foam cells and arterial plaque formation, eventually leading to massive cardiometabolic dysfunction and mortality [208]. The molecular signaling mechanisms triggered by TMAO include enhanced oxidative stress and ROS production via nucleotide-binding oligomerization domain Leucine-rich Repeat and Pyrin domain-containing 3 (NLRP3) inflammasome activation [209]. Biochemical studies have shown that TMAO-associated endothelial dysfunction occurs via high mobility group box-1 (HMGB1), an inflammatory mediator [210]. TMAO-induced CVD onset also occurs via the activation of sirtuin 3 and superoxide dismutase 2 (SIRT3-SOD2) ROS signaling. TMAO downregulates SIRT1 expression and augments oxidative stress via the p53/p21/retinoblastoma (Rb) signaling cascade [211]. TMAO also upregulates vascular oxidative stress via a rise in nicotinamide adenine dinucleotide phosphate (NADPH) oxidase activity [94] (Figure 4) [212,213]. TMAO upregulates inflammatory signaling by increasing the level of pro-inflammatory cytokines viz tumor necrosis factor (TNF)-α and IL-1β [214,215]. Augmented TNF-α levels via NF-κB signaling upregulation promotes leukocyte adhesion to endothelial walls, leading to an endothelial dysfunction that triggers thrombosis and atherosclerosis [216,217]. TMAO-associated protein kinase C activation promotes monocyte adhesion and a decrease in the self-repair capacity of the endothelium [218,219]. TMAO induces vascular cell adhesion protein 1 (VCAM1) via the methylation of NF-κB p65 subunit [134,220]. This increases monocyte adhesion through an increase in intercellular adhesion molecule 1 (ICAM1) and E-selectin, enhancing endothelial dysfunction [216,217]. TMAO also upregulates IL-6 levels, which is one of the chief promoters of foam cell formation [221,222]. TMAO plays a pivotal role in the aggregation of low-density lipoproteins (LDLs) in macrophages [122,223]. This occurs via augmenting CD36, lectin-like oxidized low-density lipoprotein receptor 1 (LOX-1) and the scavenger receptors of class A1 (SR-A1) [223]. Together, these facilitate cholesterol uptake and trigger atherosclerotic onset [224,225].

### 3.3. Bile Acids (BAs)

The gut microbiota also play a pivotal role in regulating cholesterol metabolism by altering the BA levels [226]. In the gut, the primary BAs viz cholic acid (CA) and cheno-DCA (CDCA) undergo deconjugation by the gut microbiome and bile salt hydrolase (BSH) [227,228], producing secondary BAs like DCA, lithocholic acid (LCA) and ursodeoxycholic acid (UDCA) [229]. As mentioned earlier, BAs can activate FXR, and G-protein-coupled bile acid receptor Gpbar-1-, also known as Takeda G protein-coupled receptor 5 (TGR5) [230]. Primary BAs can activate FXR to a greater extent relative to secondary BAs [231]. FXR downregulates BA uptake into the hepatocytes and augments the expression of ATP-binding cassette subfamily B member 11 (ABCB11) [232]. The plasma level ratio of secondary to primary BAs is an important marker for potential hypercholesterolemia and CAD incidence [233]. BAs regulate cardiovascular functioning by decreasing heart rate through the modulation of channel conductance and Ca^2+^ inflow in ventricular cardiomyocytes [234]. This influences vascular tone and overall cardiovascular function [235]. A reduction in secondary BA levels and a rise in primary BAs hyperactivates FXR, reduces BA production and augments CAD and hypercholesterolemia incidence [236]. Additionally, BA-induced PXR activation augments total cholesterol, very low-density lipoprotein (VLDL), and LDL, and downregulates high density lipoprotein (HDL) promoting atherosclerotic onset [237,238]. PXR activation influences the genes involved in lipoprotein transport and cholesterol metabolism, downregulating apolipoprotein A-IV (ApoA-IV) and cytochrome P-450 family 39 subfamily A member 1 (Cyp39a1) and augmenting CD36 [239]. CD36 upregulation promotes the uptake of oxidized LDL into macrophages and leads to foam cell formation, which precedes atherosclerosis [237].

### 3.4. Indole-3-Propionic Acid (IPA)

The regulation of blood pressure usually depends on mechanical capabilities of the myocardium and the peripheral vasculature [240]. A rise in blood pressure bears a pathophysiological correlation with chronic cardiac tissue injury and atherosclerosis [241]. IPA, a gut microbial metabolite of dietary tryptophan metabolism, induces myocardial contractility and the vasoconstriction of the endothelium-denuded mesenteric resistance arteries [242,243]. IPA is significantly downregulated in the serum of CAD patients [244,245]. Studies in murine models have demonstrated that dietary IPA intake attenuates atherosclerotic progression under Apolipoprotein E deficient condition [246]. IPA augments ATP-binding cassette class A1 (ABCA1) and enhances macrophage mediated reverse cholesterol transport [247], which attenuates foam cell formation and atherosclerotic onset [248,249,250,251].

### 3.5. Hydrogen Sulfide (H_2_S)

Gut-microbiota-derived H_2_S exhibits varied vascular protective functions by modulating vascular tone and blood pressure, and reducing leukocyte adhesion, platelet aggregate formation, and ROS-mediated oxidative stress [252,253,254]. H_2_S also augments endothelial cell proliferation, and decreases vascular proliferation, migration, and LDL oxidation [172]. H_2_S inhibits vascular smooth muscle cell proliferation by the downregulation of CX3CR1 expression in macrophages [255,256,257]. This occurs by promoting peroxisome proliferator-activated receptor (PPAR)-γ, coupled with the downregulation of pro-inflammatory NF-κB/ICAM-1 cascade [258]. The vasodilatory impact of H_2_S is exercised through a non-endothelial dependent mechanism characterized by the activation of vascular smooth muscle potassium (K^+^) channels [259,260], which in turn decreases intracellular pH and acidification and prevents atherosclerosis.

### 3.6. Phenylacetylglutamine (PAGln)

Beyond augmenting CVD risk in type-2 diabetes patients, PAGln has also been reported to bear a strong correlation with an increased risk of heart attack, myocardial infarction and consequent mortality [175,261,262]. In vivo studies suggest that PAGln fosters thrombus formation and a shorter time for blood flow cessation in a carotid artery injury mice model [175,263]. Clinical cohort studies suggest that PAGln augments heart-failure-associated biological activities with extensive PAGln exposure associated with sarcomere functional defects [264]. Pharmacological gain-of-function and loss-of-function studies confirm that PAGln chiefly interacts with α (α2A, α2B) and β (β2)-adrenergic receptors and influences cardiac and platelet functions that eventually culminate to atherosclerotic onset [262,265,266].

Thus, various gut microbial metabolites like SCFAs, TMAO, BAs, IPA and H_2_S play a key role in the cellular and molecular signaling responsible for modulating the CVDs’ onset and progression (Table 1).

## 4. Therapeutic Perspectives

The role of gut microbial metabolites in CVD onset and/or attenuation has gained increasing appreciation over this decade. Various strategies to efficiently manipulate these metabolite levels to ameliorate the onset and progression of heart failure, atherosclerosis, and other CVD types have been reported, as mentioned below.

### 4.1. Dietary and Lifestyle Modifications

Dietary modifications to maintain and improve gut microbial dynamics have been clinically advocated [267,268,269]. Prebiotics constitute the non-digestible carbohydrates that significantly influence gut microbiome composition and function [270]. Investigative studies revealed that regular exercise, the intake of prebiotics and/or probiotics, and associated lifestyle improvements increases the beneficial gut microbial population and decreases the propensity for cardiac fibrosis and hypertrophy [271]. Preclinical studies revealed that prebiotics empower the gut microbiota to retain its dynamics and produce metabolites responsible for cardioprotective functions [272]. This has also promoted the practice of individualized gut-microbiome-targeted approaches to provide new therapeutic strategies against cardiometabolic complications [273]. A high-salt diet reduces *Lactobacillus* abundance in the gut and augments T helper 17 cell population, resulting in the onset of salt-sensitive hypertension [274,275]. Geographical comparisons reveal that the Western diet bears greater proximity with CVD incidence compared to that of Mediterranean regions [276]. The Mediterranean diet comprises a lower level of saturated fatty acids, salts, and phosphate, and higher amounts of antioxidants, nitrates, and fiber [277,278], which support microbial balance and cause a reduction in oxidative stress, enhanced nitric oxide bioavailability, and antioxidant functions, leading to improved vascular and cardiac functioning [276].

### 4.2. Fecal Microbial Transplantation (FMT)

Fecal Microbial Transplantation (FMT) introduces fecal contents from healthy subjects into the gastrointestinal (GI) tract of gut-microbiota-compromised patients [279,280]. This technique has been found to be useful, especially in treating infectious episodes of *Clostridium difficile* invasion into the GI tract [281,282]. Recent courses of studies have identified FMT as a useful tool against the gut-microbiome-associated onset of CVDs [283]. FMT improves plasma triglyceride level and insulin sensitivity in its recipients [284]. This improves overall cardiometabolic health [285]. In experimental study models of autoimmune myocarditis, FMT has been shown to improve *Bacteroidetes* density and downregulate myocarditis incidence potential [285,286,287,288].

### 4.3. Pharmacological Interventions

Pharmacotherapy, targeting the composition and dynamics of the gut microbiota, largely contributes towards the attenuation of dysbiosis-associated CVD onset. Biguanides like metformin lower the risk of MI onset in type 2 diabetes patients [289]. Metformin functions by augmenting the peripheral glucose uptake and regulation of the incretin pathway through increasing the plasma and pancreatic levels of GLP-1, the chief molecule driving SCFA-associated cardioprotective function [290,291]. Metformin also downregulates BA absorption through hindering the apical sodium-dependent bile acid transporter (ASBT) [292], thus interfering with primary BA-mediated FXR activation [293,294]. Additionally, metformin augments the SCFA-producing *Butyrivibrio*, *Bifidobacterium bifidium*, and *Megasphera* populations [295,296].

α-glucosidase inhibitors (α-GIs), like acarbose, constitute another group of gut-microbiota-influencing drugs that reduce CVD risk in diabetic patients [297]. α-GIs upregulate bile salt hydrolase (BSH), activating the *Lactobacillus* and *Bifidobacterium* populations and downregulating the *Bacteroides*, *Alistipes* and *Clostridium* populations, thereby producing a positive impact on cardiometabolic homeostasis [298]. The pharmacological inhibition of Dipeptidyl Peptidase-4 (DPP-4) downregulates cardiovascular risk factors [299]. DPP-4 inhibition attenuates high-fat-diet-induced dysbiosis—primarily promoting the SCFA-releasing gut bacterial populations and decreasing the *Firmicutes* to *Bacteroidetes* ratio [300,301].

### 4.4. Targeting NLRP3 Inflammasomes

The NLRP3 inflammasome acts as a sensor to deleterious exogenous and endogenous entities to activate pro-inflammatory signaling and culminate in CVDs viz atherosclerosis [302,303]. Studies over the years have suggested that NLRP3 inflammasome is a key predisposing factor for atherosclerosis onset, mediated via cholesterol crystals [304]. Pharmacological studies along these lines suggest that TMAO is a significant triggering factor for NLRP3 inflammasome activation and subsequent endothelial hyperpermeability, endothelial barrier dysfunction and cardiac fibrosis [209,305].

The targeted downregulation of the NLRP3 inflammasome has been advocated across studies to attenuate atherosclerosis onset [306]. Statins, structural analogs of HMG-CoA reductase inhibitors, have been instrumental towards attenuating cardiac hypertrophy, cardiomyocyte apoptosis, endothelial dysfunction and oxidative stress [307,308]. Mechanistic studies in recent years suggest that statins impede TLR4/MyD88/NF-κB signaling which is also associated with NLRP3 inflammasome activation [309]. The suppression of NLRP3 inflammasome via Rosuvastatin has been implicated in the treatment of diabetic cardiomyopathy [310]. Simvastatin has also been implicated in hyperglycemia-associated endothelial dysfunction, downregulating vascular endothelial hyperpermeability and enhancing the expressional levels of tight and adherence junction proteins [311]. This, in turn, prevents NLRP3 inflammasome-mediated HMGB1 release in aortic endothelial cells [311]. Beyond statins, the use of Arglabin, a natural NLRP3 inflammasome inhibitor, has been found to exert anti-atherogenic effects in high-fat-diet mice model studies [312]. Together, these approaches strongly point towards NLRP3 inflammasome as a promising target for attenuating CVD onset and progression.

## 5. Current Limitations and Future Perspectives

Over the years, gut microbiota associated dysbiosis has been the focus of designing therapeutic strategies against CVD risk. Pharmacological interference, FMT, and dietary modifications have achieved considerable success in decreasing dysbiosis-induced CVD onset. However, existing limitations across these studies maintain a significant scientific gap and impede a clear understanding of the dysbiosis-associated CVD pathophysiological status.

From a pharmacological perspective, the cardioprotective impact of metformin in non-diabetic patients remain largely obscure [313]. A double-blinded placebo-controlled study with a small sample size postulates that metformin potentially improves vascular function and reduces ischemic risk in angina patients [314]. However, pilot clinical studies have mentioned no significant impact of metformin on other surrogate markers for CVD, like carotid intimal media thickness [315,316]. Similarly, the cardioprotective function of α-GIs in non-diabetic subjects remains debatable. Along these lines, studies reported that CAD patients with impaired glucose tolerance experienced no significant impact on cardiometabolic upliftment upon acarbose administration [317]. The inhibition of sodium glucose co-transporter 2 (SGT2) reduces cardiovascular inflammation, cellular apoptosis, and mitochondrial dysfunction [318]. However, its impact on gut microbial homeostasis remains largely unclear [319,320,321].

FMT constitutes an intriguing therapeutic strategy against gut-microbial-dysbiosis-driven extra-intestinal complications [284]. However, its utility remains largely restricted, owing to the associated risk of endotoxin and/or infectious substance transfer from the donor to the recipient [322,323]. The dietary intake of prebiotics strengthens gut microbial dynamics against CVD onset. However, such non-digestible carbohydrates increase the free sulfate concentrations in the colon [324], disrupting the mitochondrial complex IV function which, in turn, decreases butyrate oxidation and oxygen consumption [325]. Moreover, the emulsification property of these polysaccharides disturbs the gut microbial composition and augments bacterial translocation across the epithelium [326,327]. Considering the limitations of the existing strategies, it is plausible to say that it is important to carry out further studies to fill in the knowledge gaps in this area.

Gut microbial metabolites play a vital role in the onset as well as the attenuation of CVD, largely depending on the biochemical characteristics of the metabolite. In view of the beneficial metabolites and their cardioprotective impact, clustered regularly interspaced short palindromic repeats (CRISPR)/Cas9 technology can be advocated to alter the metabolic flow and ensure the increased and targeted expression of the cardioprotective metabolites [328,329]. Bacterial metagenomic and proteomic analysis can also be potentially combined with human metabolome to better understand the gut-microbiota-driven metabolic changes in CVD onset [330,331]. To confront the limitations associated with FMT, a transplantation of a specific group of bacterial population can be undertaken, instead of the fecal matter transplantations carried out so far [332]. From a pharmacological perspective, the development of greater numbers of cytostatic agents needs to be focused upon, instead of cytotoxic drugs, to ensure the gut microbial steady state and target specificity [333].

## 6. Conclusions

Several studies over the last few decades have proven the pivotal role of gut microbiome and its metabolites in cardiovascular health and disease, which has enabled a better understanding of the physiological and mechanistic alterations they drive in CVD onset and progression. The gut microbial breakdown of dietary food components produces a large variety of bioactive metabolites viz SCFAs, BA, TMAO, IPA, phenolic compounds, and hydrogen sulfide, among many. Precision medicine-based studies have promoted personalized, integrated and patient-specific approaches for CVD therapy. In this milieu, examining the microbiome of individual patients and analyzing their gut metabolites in plasma could provide us with necessary information to identify the detrimental effects of gut metabolites on cardiovascular health and equip us with personalized treatments. An in-depth comprehension of the signaling cross-talks involved in the gut–heart axis and alternative strategies to improve the existing therapeutic courses in relation to gut microbial metabolites shall potentially benefit the development of effective druggable targets and mitigate CVD over the long term.

## Figures and Tables

**Table 1:** A summary of the source, mechanism of action and CVD impact of key gut microbial metabolites associated with CVD onset and progression.

Metabolite	Source	Mechanism of Action	Impact on CVDs	References
Short-Chain Fatty acids (SCFAs)	Gut microbial fermentation of dietary macromolecules	Block cholesterol generation and their hepatic localization, promote Treg-induced anti-inflammatory signaling and reduce the local infiltration of immune cells, promote the expression of protein phosphatases, eNOS, PTEN and GSK-3β	Downregulate cardiac hypertrophy, fibrosis and associated vascular dysfunction, inhibit onset of coronary diseases, reverses cardiomyocyte contraction defects	[185,193,196,198]
Trimethylamine-N-oxide (TMAO)	Gut-microbiota-driven breakdown of high-fat dietary food components via microbial TMA lyases	Enhances oxidative stress and ROS production via NLRP3 inflammasome activation, downregulates SIRT1 levels and promotes oxidative stress via the p53/p21/retinoblastoma (Rb) signaling, upregulates vascular oxidative stress via an upsurge in NADPH oxidase activity	Promotes monocyte adhesion and deteriorates endothelium self-repair capacity, upregulates foam cell formation, augments macrophage LDL aggregation and is cumulatively predisposed towards atherosclerosis	[214,216,217,218,219,220,225]
Bile acids (BAs)	Amphipathic molecules generated in the liver from cholesterol metabolism	Activate FXR, TGR5, modulation of Ca^2+^ inflow in ventricular cardiomyocytes, induce PXR activation, downregulating ApoA-IV, Cyp39a1 and augments CD36	Influence vascular tone and overall cardiovascular function, a reduction in secondary BA and a rise in primary BAs promotes CAD and hypercholesterolemia, increases total cholesterol, VLDL, and LDL, and decreases HDL that lead to atherosclerosis	[229,230,232,234,235]
Indole-3-propionic acid (IPA)	Gut-microbiota-driven tryptophan breakdown	Enhances ABCA1 and macrophage-associated reverse cholesterol transport	Induces myocardial contractility, the vasoconstriction of endothelium-denuded mesenteric arteries	[242,243,244,245]
Hydrogen sulfide (H_2_S)	Sulfate-reducing gut microbiota	Induces the downregulation of macrophage CX3CR1 expression, promotes PPAR-γ and downregulates NF-κB/ICAM-1	Exhibits vascular protective functions, regulates vascular tone, reduces leukocyte adhesion, mitochondrial ROS stress, decreases vascular proliferation and LDL oxidation.	[255,256,257,259,260]
Phenylacetylglutamine (PAGln)	Phenylalanine metabolism, primarily driven via bacterial PAGln synthase	Interacts and activates α2A, α2B and β2- adrenergic GPCRs	Associated with sarcomere functional defects, influences cardiac and platelet functions that eventually culminate to atherosclerosis	[175,262,263,264]

## Data Availability

No new data were created or analyzed in this study.
